# Supplementary material for: Mutations in PCYT2 disrupt etherlipid biosynthesis and cause a complex hereditary spastic paraplegia
Source: Brain. 2019 Oct 22;142(11):3382–97. doi: 10.1093/brain/awz291 (PMC6821184; doi:10.1093/brain/awz291)
Supplement: awz291_Supplementary_Data [file awz291_supplementary_data.zip › awz291-Suppl_data/Supplementary_Data4.zip!/Supplementary information 3 - fibroblast lipidomics.zip!/Fibroblast lipidomics/Controle vs Patient2/Statistical plots and data/SM[t] species/Volcano plot_alternative.pdf]

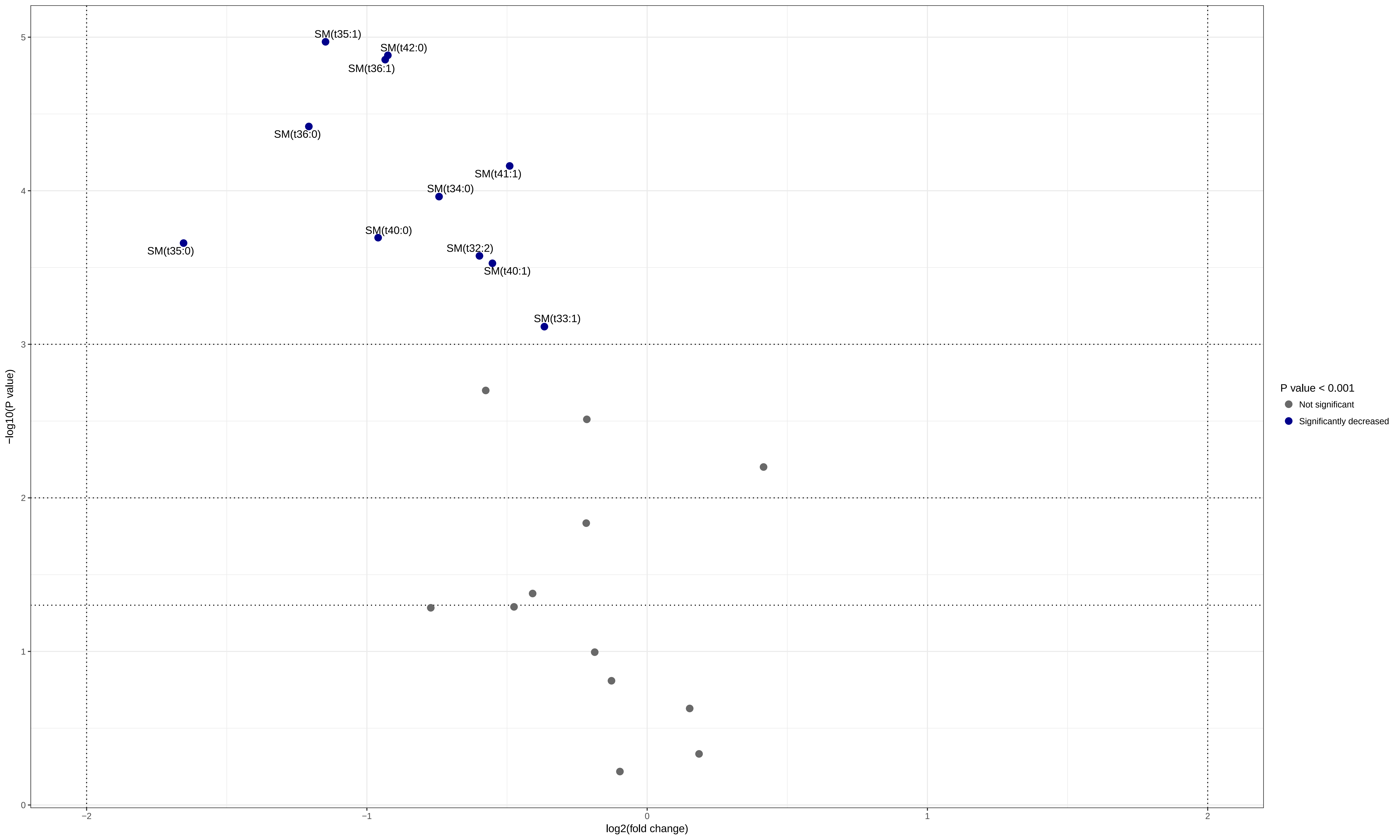

SM(t35:1)
SM(t42:0)
SM(t36:1)
SM(t36:0)
SM(t41:1)
SM(t34:0)
SM(t40:0)
SM(t35:0)
SM(t32:2)
SM(t40:1)
SM(t33:1)
5
4
3
2
1
0
−2
−1
0
1
2
−log10(P value)
log2(fold change)
P value < 0.001
Not significant
Significantly decreased
